# The Cerebrospinal Fluid in Multiple Sclerosis

**DOI:** 10.3389/fimmu.2019.00726

**Published:** 2019-04-12

**Authors:** Florian Deisenhammer, Henrik Zetterberg, Brit Fitzner, Uwe K. Zettl

**Affiliations:** ^1^Department of Neurology, Innsbruck Medical University, Innsbruck, Austria; ^2^Department of Psychiatry and Neurochemistry, Institute of Neuroscience and Physiology, The Sahlgrenska Academy at the University of Gothenburg, Mölndal, Sweden; ^3^Clinical Neurochemistry Laboratory, Sahlgrenska University Hospital, Mölndal, Sweden; ^4^Department of Neurodegenerative Disease, UCL Institute of Neurology, London, United Kingdom; ^5^The Fluid Biomarker Laboratory, UK Dementia Research Institute at UCL, London, United Kingdom; ^6^Division of Neuroimmunology, Department of Neurology, University Medicine Rostock, Rostock, Germany

**Keywords:** CSF (cerebrospinal fluid), biomarker, multiple sclerosis, oligoclonal band (OCB), neurofilament light (NfL)

## Abstract

Investigation of cerebrospinal fluid (CSF) in the diagnostic work-up in suspected multiple sclerosis (MS) patients has regained attention in the latest version of the diagnostic criteria due to its good diagnostic accuracy and increasing issues with misdiagnosis of MS based on over interpretation of neuroimaging results. The hallmark of MS-specific changes in CSF is the detection of oligoclonal bands (OCB) which occur in the vast majority of MS patients. Lack of OCB has a very high negative predictive value indicating a red flag during the diagnostic work-up, and alternative diagnoses should be considered in such patients. Additional molecules of CSF can help to support the diagnosis of MS, improve the differential diagnosis of MS subtypes and predict the course of the disease, thus selecting the optimal therapy for each patient.

## Introduction

Oligoclonal bands (OCB) of the cerebrospinal fluid (CSF) have been important in the diagnosis of multiple sclerosis (MS) for many years. The further search for biomarkers is of great importance in order to improve the diagnosis and therapy of MS. This review is divided into 2 parts. The first part focuses on OCB as diagnostic biomarker for MS and briefly describes other diagnostic markers such as aquaporin4 (AQP4) and biomarkers that are about to enter clinical routine, such as anti-myelin oligodendrocyte glycoprotein (MOG). The second part is about CSF molecules, which have been described in research as potential biomarkers.

## Part I: The Clinical Laboratory

### Cerebrospinal Fluid—General Considerations

Whenever investigations are required either to make or rule out a particular disease, it is of utmost importance to know what one would normally expect from such an investigation, i.e., to have access to normal or reference values. This goes of course also for clinical chemistry tests performed in CSF. As a prerequisite for making reference values global and assay-independent, it is important to standardize the field through the certification of reference methods and materials that can be used as external calibrators for assay manufacturers. It is also important to establish external quality control programmes to make sure laboratories are both accurate and precise. Internal stability of the measurements also has to be monitored using internal control samples each time a test is performed.

It is surprising how little progress has been made in the field of reference values for CSF analytes since the first systematic assessment of CSF normal values by Meritt and Fremont-Smith (1). For one of the most basic CSF variables, i.e., total protein and albumin, normal values based on modern quality standards have been evaluated and published only recently (2). Most labs adopt historical reference values without validating their own (3). Even if normal values have been established in some labs the methods of evaluation suffer from methodological shortcomings, such as selection bias, poor definition of normal cohorts, and statistical errors (2). Because upper reference limits for total CSF protein are mostly too low it has been estimated that approximately in 15% of normal CSFs total protein values are falsely reported as pathologically elevated. Similar issues have been found with CSF glucose measurements and formulas for intrathecal immunoglobulin synthesis (4, 5). Glucose measurements must be done in CSF and serum simultaneously and a ratio needs to be calculated. The glucose ratio cut-off values depend on serum glucose levels because the transporter systems across the blood-brain-barrier (BBB) have limited capacities. This fact is often not considered by CSF labs. For intrathecal synthesis of immunoglobulins it is well-known that the widespread Reiber-formula overestimates particularly intrathecal IgM and IgA synthesis rates (4, 6).

### How Is All This Related to the Diagnosis of MS?

Because the etiology and specific pathogenesis of MS are unknown, there is no specific test, be it lab-based or otherwise, available. In diseases with a known cause, e.g., infections, a specific test detecting the infectious agent or antibodies against it is most frequently available. Even in entities in which the cause is not fully elucidated but the pathomechanism is evident, such as autoimmune encephalitides, a specific test detecting the auto-antibody can be used to make the diagnosis (7). In MS there is no such specific test available which is why one needs to rely on “circumstantial evidence.” The diagnosis is based on typical, yet not limited to, clinical findings, magnetic resonance imaging (MRI), and CSF as well as other investigations (8). Doctors are well-advised to use all these tools in order to optimize the diagnostic accuracy.

In the past two decades the diagnostic criteria for MS have been updated 4 times (8–11). Starting with the revision in 2001 (9) CSF was less and less required to confirm the diagnosis in the subsequent updates until 2010 (11). As some authors suspected (12), ignorance of diagnostic tools might have led to insufficient diagnostic performance, in that the rate of MS misdiagnosis increased, even though there is no formal proof that this phenomenon occurred due to the decrease in CSF examinations (13). Mostly, misdiagnosis was due to overinterpretation and misinterpretation of MRI findings (13). Moreover, the true diagnoses were most often migraine, fibromyalgia, unspecific symptoms, or psychogenic disorders (14). In these diagnoses, CSF findings are usually normal, including markers of intrathecal immune-activation such as quantitative elevation of immunoglobulins (e.g., IgG-index) or detection of OCB. One must keep in mind that the negative predictive value of OCB in neurological patients who had undergone LP was 90% (15), and even in patients with clinically isolated syndromes (CIS—a clinical syndrome highly suspicious of a first manifestation of MS) the negative predictive value of OCB was 88% (16). So, the lack of OCB in CSF must be considered a red flag in the differential diagnostic work-up. In this context, it should be remembered that the first reported case of natalizumab-associated progressive multifocal leukoencephalopathy occurred in a very likely misdiagnosed patient, who had no detectable OCB in CSF in two consecutive occasions (17). In fact, the vast majority of misdiagnosed patients get actually treated with MS drugs (14).

### Oligoclonal Bands in CSF—How Likely Is It MS?

It is well-known that OCB in CSF are not exclusively found in MS. OCB are thought to indicate chronic immune-activation in the CNS and therefore, can be found in a variety of chronic inflammatory diseases. The positive predictive value (PPV) of OCB for MS depends on the control or reference population—an inherent issue with PPV—and on the integration of other CSF findings, such as cell counts or albumin/protein concentrations. E.g., in neuroborreliosis, OCB are frequently encountered, in contrast to MS, however, total protein concentration and CSF cell counts are substantially higher (18). Several authors found OCB in CSF highly sensitive and specific for MS (19), which is likely due to the fact that other diseases with OCB in CSF occur relatively seldom. However, when inflammatory diseases are particularly considered, the specificity of OCB for MS drops substantially from 94 to 61%, as shown in a meta-analysis (20). This highlights again that the diagnostic tools for MS are not uni-dimensional.

Apart from MS, there is a long list of diagnoses with CSF OCBs reported: systemic lupus erythematosus, neurosyphilis, neurological paraneoplastic disorders, Behcet's disease neuroborreliosis, aseptic meningitis, neurosarcoidosis, HIV infection, cerebral tumors including lymphomas, Sjögren's syndrome, herpes encephalitis, Morvan syndrome, Anti-NMDA and other autoimmune encephalitis, neurotuberculosis, anticardiolipin syndrome, HTLV myelopathy, prion disease, schistosomiasis, stiff-person syndrome, cerebral cysticercosis, GBS, CNS vasculitis (20). One must be careful however, in our experience running a clinical CSF lab for decades, we rarely detected OCB in solid cerebral tumors, prion disease, or GBS for instance.

### Some Methodological Considerations

As outlined above, a proper assessment of normal and reference values should be done in each CSF lab rather than adopting such values from the literature. Also, validation in case of in-house developed assays must be done, or at least verification in case of commercially available, externally validated tests (21). One of the key CSF tests in query MS including differentials is the method of isoelectric focusing (IEF) (22). This method has been developed in the 70ies and has since then undergone several refinements. Today, IEF followed by IgG specific immunoblot is the recommended standard for detection of OCB (19). These guidelines developed some essential rules for CSF IgG detection as shown in Table 1. Importantly, intrathekal IgG synthesis can only be assessed if compared to serum. OCB in CSF can only be considered intrathecally synthesized if the bands selectively occur in CSF or if there are more bands in CSF than in serum, referred to as pattern 2 and 3 according to Freedman et al. (19). Depending on the IgG separation method, serum bands should be outnumbered by 1–3 bands in CSF (23). Identical bands in CSF and serum do not reflect pathological immunoglobulin synthesis in the CNS because the CSF bands have their origin in the systemic circulation. These findings are referred to as pattern 4 (identical oligoclonal) and 5 (identical monoclonal) according to Freedman et al. (19).

**Table 1:** Guidelines for IgG detection in CSF according to Freedman et al. (19).

CSF immunoglobulins must be separated by IEF
CSF immunoglobulins must not be separated by electrophoresis
CSF must not be concentrated
CSF immunoglobulins should be immunofixed/blotted
CSF and parallel serum must have similar amounts of immunoglobulin on the same analytical run
IEF is always more sensitive than any quantitative formula for immunoglobulins in CSF/serum
To use “only” a quantitative formula is not recommended
Non-linear formulations are recommended over linear formulations
A quantitative formula may be more useful in treatment/prognosis than in diagnosis
Light chain immunofixation can extend the value of IgG immunofixation


More recent developments regarding measurements of intrathecal immune activation include detection of free light chains (FLC). There are several reports that, particularly, kappa FLC are equally sensitive and specific for clonal expansion as detection of OCB in MS (24). The advantages of FLC measurements are its methodological simplicity and its objective read-out by instrumental measurements of concentrations rather than visual inspection of OCB. However, before general implementation of FLC detection or even replacement of IEF there is more work needed including independent confirmation by different labs and validation of specificity using broader ranges of control groups, particularly other inflammatory diseases.

A comprehensive overview regarding methodological aspects of CSF investigations in general can be found in recent publication (23).

### Expected CSF Changes in MS

**Figure 1:**
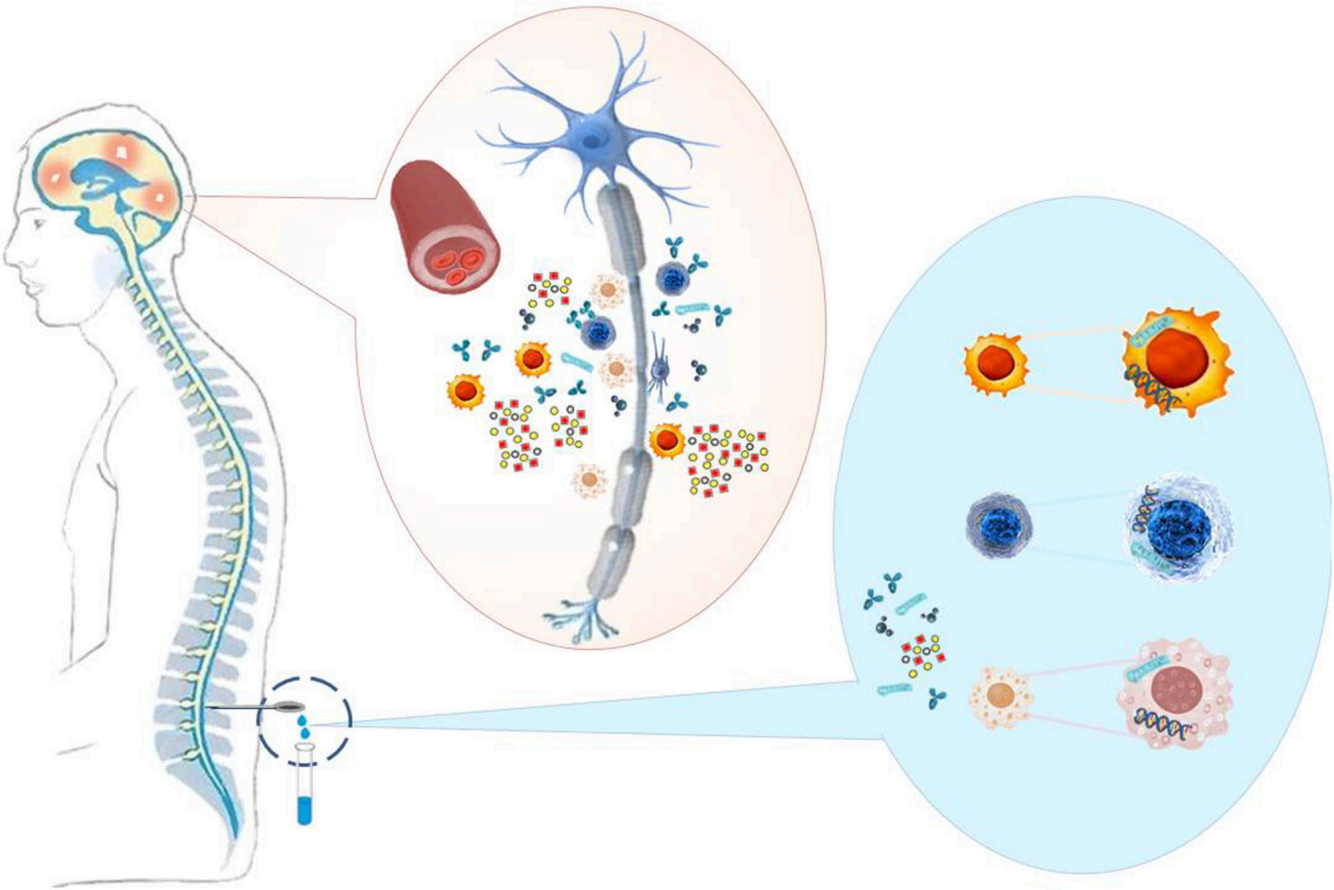
MS causes neuronal damage (demyelination, axon degeneration, synaptic loss) to the brain and spinal cord. Immune cells, pathological antibodies, adhesion molecules, cytokines, chemokines, and nucleic acids, which reflect inflammations in the CNS, are present in the CSF of the patients and can serve as biomarkers to support MS diagnosis and therapy.

As MS is considered an inflammatory CNS disease with focal breakdown of the BBB one could expect markers of these events in CSF to be altered (Figure 1). Markers of these changes are CSF leukocyte counts as an indicator of inflammation (apart from elevated immunoglobulin levels), and total protein or albumin concentrations as an indicator of BBB disruption (23) (Table 2).

**Table 2:** CSF changes in MS.

**CSF variable**	**Expected finding**
Total protein/albumin quotient	Normal, rarely slightly elevated
CSF:serum glucose ratio	Normal
CSF leukocyte count	Mild pleocytosis in 50% of patients Less than 50 cells/uL in 98%
Cytology	Dominated by lymphocytes (90%), some monocytes. Rarely macrophages, plasma cells, granulocytes
Immunoglobulins quantitative	IgG concentration by linear or non-linear formulae elevated in 60–70% of patients, IgA and IgM synthesis may be found less frequently
Immunoglobulins qualitative	Oligoclonal bands in 95% of definitive MS cases, 85% in CIS

In about one half of MS patients CSF leukocyte counts will be elevated up to 50 cells per uL (22). Higher leukocyte counts occur in only 1–2% of patients and should give raise to consider alternative diagnoses, particularly infectious CNS diseases. On differential cell count lymphocytes dominate by far, accounting for more than 90% of cells, 90% of which are T-cells and 10% B-cells, which excludes lymphocyte subtyping as a distinctive feature of MS (25). The remainder is constituted by monocytes although other leukocyte types may be encountered such as plasma cells, macrophages, and very rarely granulocytes. Again, a substantial deviation from this pattern should be regarded as red flag regarding the correctness of the diagnosis.

Glucose CSF to serum ratios are normal in MS (26).

Total protein or albumin quotient is normal in the vast majority of patients (22, 27), which is in line with the very focal and transient BBB leakage in MS.

The hallmark of typical CSF changes in MS however, is the increased production of intrathekal immunoglobulins (28). To demonstrate this, the MS diagnostic guidelines refer to two different methods: first, quantitatively elevated IgG as shown by e.g., the IgG index, and second, detection of OCB by IEF (9). It must be kept in mind that any quantitative formula is less sensitive than OCB detection with elevated IgG being found in ~60% of MS patients compared to 95% being OCB positive (i.e., diagnostic sensitivity) (19, 29). Even though it is not an MS specific test, the diagnostic specificity lies between 61 and 93% depending on the reference group (30). The lowest specificity rates occur if other inflammatory CNS diseases are exclusively included in the comparator group. In a mixed reference population, one would expect the diagnostic specificity to be probably in the middle of these values, which means that OCB have a very acceptable diagnostic performance comparable to, e.g., amyloid-beta and tau proteins in Alzheimer's disease (30).

Apart from a diagnostic role OCB are of prognostic value in CIS patients with a hazard ratio of 2.18 (95% confidence interval: 1.71–2.77) for the prediction to convert to clinically definite MS (31). A fact that has been described for conversion to MS after optic neuritis 20 years ago (32).

Given the inflammatory process, MS patients also have increased concentrations of a number of cytokines, chemokines, and interleukins in their CSF, e.g., CXCL13, IL6, IL8, and IL10 (33).

### CSF Findings in Other Inflammatory Demyelinating Diseases

At first onset some symptoms are similar between MS and other inflammatory demyelinating diseases, particularly neuromyelitis optica (NMO) spectrum diseases. These syndromes can be diagnosed by IgG antibodies against AQP4 or MOG (34). In general CSF work-up there is a distinct feature, which is a lack of CSF OCB in NMO spectrum diseases in 80–90% of patients (35). Total leukocyte counts in NMO spectrum disorders are similar to MS with pleocytosis being found in around 50% of patients, exceeding rarely 100 cells per uL (36). However, on differential cell counts granulocytes occur somewhat more frequently in NMO spectrum disorders compared to MS (36).

In MOG-IgG antibody associated syndromes the frequency of OCB of 13% is similarly low as in NMO spectrum disorders (37). It seems however, that CSF pleocytosis occurs more frequently, i.e., in almost two thirds of patients with a relatively high proportion of neutrophils making up 22% of all leukocytes (37). Also, an elevated albumin quotient can be found in roughly one third of patients with MOG IgG antibodies, particularly if spinal symptoms occur.

Altogether, the main distinctive feature between these syndromes and MS is the frequency of OCB, whereas general CSF changes (i.e., cell counts, cytology, protein) differ slightly but do not provide compelling evidence for or against one of the entities.

## Part II: The Research Laboratory

### Spectrum of Biomarkers in CSF

MS is an inflammatory disease characterized by damage and repair processes. The search for biomarkers focuses not only on cells and molecules of the immune response, but also on molecules reflecting the heterogeneity of mechanisms involved in the disease. Many findings on potential biomarkers have been published, including antibodies, cytokines, and chemokines molecules involved in damage and repair processes, proteins of the complement system as well as nucleic acids, that could help in MS diagnosis, differential diagnosis, prognosis, and in disease or therapy monitoring. In Table 3 we listed information on various biomarkers mentioned in this article. Of these biomarkers, neurofilament light (NfL) is currently one of the most promising.

**Table 3:** Selection of molecular and cellular markers and their potential utility in MS diagnosis, prognosis and monitoring.

	**Diagnosis**		**Prognosis (risk factor for)**		**Monitoring**		
	**CDMS**	**NMOSD**	**CDMS**	**Worse disease course**	**Therapy effects**	**Therapy side effects**	**References**
**ANTIBODIES**							
Anti-AQP4		↑^4, 6, 8*^					(38, 39)
Anti-JCV						↑^a^	(40, 41)
Anti-MOG					↓^f^		(42–44)
OCB	↑^4^	↓^6^	x				(31, 32, 35, 39, 45, 46)
OCGB	↑^1^		x	x	↓^a^		(39, 47–51)
OCMB	↑^4, 3, 5^		x	x			(39, 51–57)
**CYTOKINES/CHEMOKINES**							
CXCL13 (SDF-1α)	↑^9^		x	x	↓^a,b,c,g^		(33, 39, 51, 58–66)
IL-6		↑^4, 6^			↓^a^		(33, 39, 60, 67, 68)
IL-8					↓^a^		(33, 60)
**DAMAGE AND REPAIR MOLECULES**							
14-3-3	↑^2^		x	x			(39, 69, 70)
CHI3L1	↑^1, 4, 7^		x	x	↓^a,d,g^		(39, 58, 59, 61, 71–74)
GFAP	↑^1^	↑^6^		x			(39, 59, 61, 64, 75–77)
Haptoglobin		↑^6^					(39)
NfH	↑		x		↓^a^		(39, 58, 59, 78–80)
NfL	↑^1, 2^		x	x	↓^a,c,d,g^		(39, 58, 59, 61, 64, 75, 77, 79–88)
**ADHESION MOLECULES**							
sICAM-1		↑^1, 2, 6^					(89–92)
sVCAM-1		↑^1, 2, 6^					(65, 89, 90, 93)
**COMPLEMENT COMPONENTS**							
C1inh		↑^6, 9^					(94)
C1s		↑^6, 9^					(94)
C5		↑^6, 9^					(94)
Factor H		↑^6, 9^					(94)
**OTHER MOLECULES**							
sCD21	↑^2^				↓^a,b^		(95, 96)
sCD27	↑^1, 2^				↓^a,b^		(95, 96)
**NUCLEIC ACIDS**							
JCV DNA						↑^a,c,e,g^	(97, 98)

Findings of molecular markers in MS-specific clinical contexts are listed in the table. An arrow pointing upwards indicates an elevation and an arrow pointing downwards a decrease in the amount of the respective molecule in CSF. NMOSD data were only considered when a difference to MS was described. (1) compared to HC; (2) compared to NIND; (3) compared to OIND; (4) compared to OND; (5) compared to distinct disease groups or mixtures of control groups; (6) compared to MS; (7) compared to CIS; (8) compared to RRMS (

* in remission);

(9) compared to control whose composition was not mentioned;

(a) natalizumab;

(b) steroids;

(c) B-cell depletion therapy;

(d) mitoxantrone;

(e) dimethylfumarate;

(f) DNA plasmid vaccine BHT-3009;

(g) *fingolimod*.

### CSF and Serum NfL as a Biomarker of Disease Intensity in MS

Research over the past three decades have revealed that increased CSF concentration of the axonal injury marker NfL reflects disease activity and progression in all forms of MS (81). It has also become clear the concentrations dynamically change in response to relapses and treatment; MS patients starting natalizumab, a disease-modifying therapy (DMT) with high efficacy, experienced a normalization of their CSF NfL levels down to those seen in healthy controls within 6–12 months (82), suggesting that NfL can be used to monitor therapeutic efficacy. Similar observations have been made for fingolimod in patients with relapsing remitting (RR) MS and for mitoxantrone or rituximab and natalizumab in progressive MS (81). Recent ultrasensitive assays have made it possible to measure the biomarker in blood (serum or plasma; either matrix works fine), showing excellent correlation with CSF (99). Blood NfL behaves similar to CSF, also in response to DMTs, making it a promising blood biomarker for monitoring of treatment efficacy (100, 101). Ongoing studies are now also exploring it as a potential biomarker to detect side effects and suboptimal treatment efficacy. A limitation of CSF and blood NfL is that the marker is not specific to any diagnosis; it is a general marker of axonal injury and increases in all neurological disorders that involve such a process (81).

### Areas of Application for CSF Biomarkers

#### Diagnosis

For a more reliable diagnosis of MS, many studies focus on changes in CSF composition to find markers that distinguish between MS and neuronal diseases with similar symptoms. Recently, antibodies against aquaporin 4 (AQP4) were identified in CSF of NMO, but not in MS patients (38, 39) (Table 3). Since these antibodies are not present in every NMO patient, additional markers are needed. Another newly discovered biomarker is the anti-MOG antibody found in the CSF of patients with demyelinating diseases such as optic neuritis (usually recurrent), myelitis encephalitis, brainstem encephalitis, and acute disseminated encephalomyelitis (ADEM)-like presentations. Today, MOG-IgG-associated encephalomyelitis (MOG-EM) is considered a separate disease entity (34).Other candidates of potential biomarkers are described in the group of cytokines [e.g., interleukin (IL)-6] (39), adhesion molecules [such as soluble intracellular and vascular cell adhesion molecule (sICAM and sVCAM) (89)], damage and repair associated molecules [like glial fibrillary acidic protein (GFAP) and haptoglobin] (39) and complement components [e.g. Complement component 1-inhibitor (C1inh), C1s, C5 and factor H] (94) (Table 3). Further studies need to evaluate the benefit of these molecules in diagnosis.

#### Prognosis

Prognostic CSF markers may influence the choice of therapy for MS, for example, when it is possible to distinguish between a very active disease course and a mild progression. Protein chitinase 3-like1 (CHI3L1) and NfL are today the most promising prognostic CSF markers to predict conversion of MS on the one hand and disability on the other (58). Other markers that have been shown to have prognostic potential for predicting the conversion of CIS to clinical definite (CD) MS, from RRMS to secondary progressive (SP) MS and a worse disease progression include oligoclonal IgM bands (OCMB) and protein 14-3-3 (39).

#### Monitoring of Therapy Response and Side Effects

For MS various DMTs are approved by EMA and FDA. Different CSF markers are described in particular molecules of neuronal damage, pro- and anti-inflammatory cytokines and chemokines, as well as damage and repair molecules that are influenced by DMTs and that may reflect the efficacy of therapy (Table 3). Treatment with Natalizumab, for which most data on CSF molecules are available, leads, besides a decrease of NfL, to a downregulation of CHI3L1, neurofilament heavy (NfH), IL-6, IL-8, and chemokine (c-x-c motif) ligand CXCL13 (33, 39, 82, 102) in CSF (Table 3). CXCL13 is also downregulated in CSF of MS patients treated with steroids, B-cell depletion therapy or fingolimod (39, 59). CHI3L1 is down-regulated in CSF of MS patients not only by natalizumab but also by treatment with fingolimod and mitoxantrone (39, 59). Thus, both molecules could serve as markers for therapy-response, CXCL13 as marker of anti-inflammatory drugs and CHI3L1 for monitoring the decrease in cell damage. Recently, elevated levels of soluble cluster of differentiation (sCD) 27 and sCD21 have been found in the CSF of MS patients (95) and, in particular, sCD27 has been highlighted as a therapeutically responsive (natalizumab and methylprednisolone) potent and sensitive marker for intrathecal inflammation in progressive MS (96).

DMTs have been available for MS treatment for over 20 years and new DMTs with higher efficacy have been continuously developed since then. Depending on the mode of action of individual drugs, the risk of bacterial, viral, parasitic and/or fungal infection may increase (103). Existing latent viral infections can become active and trigger a severe infection under DMT, as the modulation of the immune system can lead to a decreased anti-viral immune response. Best known is the development of progressive multifocal leukoencephalopathy (PML) in MS patients infected with John Cunningham Virus (JCV) as a severe side effect of natalizumab therapy. Natalizumab is associated with the highest risk of PML (incidence: one in 250) of all approved MS therapies to our current knowledge (104–106). The frequency of PML increases with the duration of natalizumab and former JCV-negative patients may change to JCV-positive ones. Several cases of PML have also been reported in MS patients treated with fingolimod or dimethylfumarate (104–106). Although there are no known cases of PML from alemtuzumab, mitoxantrone, B-cell depletion or teriflunomide in MS patients, a risk cannot be dismissed because these drugs or closely related compounds have been associated with PML in other diseases (105). The detection of JCV infection by anti-JCV indices can be prevented by B-cell depletion therapies such as Rituximab (107), since antibody production decreases with decreasing B-cell numbers. Therefore, careful monitoring of anti-JCV antibodies and/or JCV DNA in the blood and CSF is necessary, in particular for natalizumab treatment and suspected PML (108).

Not only JCV, but also other viral infections, which can even lead to encephalitis, can occur under DMTs. The risk of severe viral infections increases with cladribine (mainly herpes zoster), ocrelizumab and natalizumab (herpes), and fingolimod (herpes and varicella). Two deaths from herpes and varicella encephalitis have been reported for fingolimod (106). For this reason, careful monitoring of MS patients treated with DMTs is recommended. If virus-induced encephalitis is suspected, DNA analyses in the CSF may be useful for diagnosis.

## Conclusions

OCB are important biomarkers that can support MRI diagnostics and help to avoid false-positive MS diagnoses. Therefore, the revised McDonalds criteria have increased the importance of the OCB.

New biomarkers such as AQP4 have now established themselves in clinical practice, and others such as Anti-MOG and NfL are about to enter clinical routine.

An important focus in the search for new biomarkers is the monitoring of therapy efficacy and the prediction of severe side effects.

Many other CSF molecules such as CHI3L1, IL-6, or CXCL13 show potential as markers for clinical practice, but further research is needed to prove their importance.

## Author Contributions

All authors listed have made a substantial, direct and intellectual contribution to the work, and approved it for publication.

### Conflict of Interest Statement

The authors declare that the research was conducted in the absence of any commercial or financial relationships that could be construed as a potential conflict of interest.

## Abbreviations

AQP4: aquaporin 4
C1inh: Complement component 1-inhibitor
CAM: cell adhesion molecule
CDMS: clinical definite MS
CSF: cerebrospinal fluid
CHI3L: protein chitinase 3-like
CIS: clinically isolated syndrome
CXCL: chemokine (c-x-c motif) ligand
GFAP: glial fibrillary acidic protein
HC: healthy control
IL: interleukin
JCV: John Cunningham virus
MOG: myelin oligodendrocyte glycoprotein
MS: multiple sclerosis
Nf: neurofilament
NfH: Nf heavy
NfL: Nf light
NIND: non-inflammatory neurological disease
NMOSD: neuromyelitis optica spectrum disorders
OCB: oligoclonal bands
OCGB: oligoclonal immunoglobulin G bands
OCMB: oligoclonal immunoglobulin M bands
OIND: other inflammatory neurological disease
OND: other neurological disease
RRMS: relapsing-remitting MS
sCD: soluble cluster of differentiation
sICAM: soluble intercellular CAM
sVCAM: soluble vascular CAM.
